# Changing the Face of Kynurenines and Neurotoxicity: Therapeutic Considerations

**DOI:** 10.3390/ijms16059772

**Published:** 2015-04-29

**Authors:** Zsuzsanna Bohár, József Toldi, Ferenc Fülöp, László Vécsei

**Affiliations:** 1 MTA-SZTE Neuroscience Research Group, Semmelweis u. 6, Szeged H-6725, Hungary; E-Mails: zsuzsanna.bohar@gmail.com (Z.B.); toldi@bio.u-szeged.hu (J.T.); 2 Department of Physiology, Anatomy and Neuroscience, Faculty of Science and Informatics, University of Szeged, Közép fasor 52, Szeged H-6726, Hungary; 3 Institute of Pharmaceutical Chemistry, Faculty of Pharmacy, University of Szeged, Eötvös utca 6, Szeged H-6720, Hungary; E-Mail: fulop@pharm.u-szeged.hu; 4 Department of Neurology, Faculty of Medicine, Albert Szent-Györgyi Clinical Center University of Szeged, Semmelweis u. 6, Szeged H-6725, Hungary

**Keywords:** kynurenines, neurotoxicity, neurodegeneration

## Abstract

Kynurenines are the products of tryptophan metabolism. Among them, kynurenine and kynurenic acid are generally thought to have neuroprotective properties, while 3-hydroxykynurenine, 3-hydroxyanthranilic acid and quinolinic acid are considered neurotoxic. They participate in immunoregulation and inflammation and possess pro- or anti-excitotoxic properties, and their involvement in oxidative stress has also been suggested. Consequently, it is not surprising that kynurenines have been closely related to neurodegenerative diseases, such as Alzheimer’s disease, Parkinson’s disease, Huntington’s disease, amyotrophic lateral sclerosis and multiple sclerosis. More information about the less-known metabolites, picolinic and cinnabarinic acid, evaluation of new receptorial targets, such as aryl-hydrocarbon receptors, and intensive research on the field of the immunomodulatory function of kynurenines delineated the high importance of this pathway in general homeostasis. Emerging knowledge about the kynurenine pathway provides new target points for the development of therapeutical solutions against neurodegenerative diseases.

## 1. Introduction

Nowadays, the interpretation of neurotoxicity is not confined to the idea of external substances causing neuronal damage. Basically, every substance or phenomenon, whether it is internal or external, causing damage to the neurons is considered neurotoxic.

Neurodegenerative diseases, such as Parkinson’s disease (PD), Alzheimer’s disease (AD), Huntington’s disease (HD), amyotrophic lateral sclerosis (ALS), multiple sclerosis (MS), *etc*., all possess characteristics related to neurotoxic processes. Consequently, neurodegeneration in general could be attributable to various forms of neurotoxicity. Neurodegenerative diseases have different courses and distinct clinical symptoms; however, they share some common mechanisms, which eventually lead to neuronal death. These processes are the imbalance in intracellular energy homeostasis, excitotoxicity and inflammation [1,2,3]. A long line of evidence proves that the kynurenine pathway (KP) of tryptophan (TRP) metabolism and the pathomechanism of neurodegenerative diseases are associated at several points [4]. This review will focus on the properties of kynurenine metabolites related to neurotoxicity also occurring in neurodegenerative diseases.

## 2. Common Neurotoxic Mechanisms in Neurodegeneration

The neural tissue is the main energy consumer of the human body, and imbalance in energy homeostasis can lead to neuronal deficit and, eventually, to neuronal death. The energy, in the form of ATP, is provided by the mitochondria. These organelles are the scenes of the citric acid cycle, fatty acid oxidation, the urea cycle and oxidative phosphorylation. Impaired mitochondrial function leads to energy deficit (lack of ATP), which, in turn, leads to the disruption of Na^+^/K^+^-ATP-ase, Ca^2+^/H^+^-ATP-ase and the reversion of the Na^+^/Ca^2+^ transporter [5]. Under these circumstances, the cells are not able to maintain their normal membrane potential, resulting in depolarization. Cells with disrupted membrane potential are more prone to excitotoxic and oxidative damage [6].

Furthermore, impaired mitochondrial function causes the uncontrolled generation of reactive oxygen (ROS) and nitrogen species (RNS): superoxide anion (O_2_^•−^), hydroxyl radical (^•^OH), hydrogen peroxide (H_2_O_2_), nitric oxide (^•^NO), nitrogen dioxide (^•^NO_2_) and peroxynitrite anion (ONOO^−^). ROS and RNS can attack macromolecules, resulting in misfolded proteins, lipid peroxidation or nitrosylation and nucleic acid damage. However, mitochondria are not the only source of ROS in the cells; peroxisomes and the endoplasmic reticulum are also capable of ROS production. Besides, numerous enzymes are known to produce ROS, too, e.g., NADPH oxidases, cyclooxygenases, xanthine oxidase, cytochrome P450 enzymes and nitrogen oxide synthases [7]. The excess amount of reactive species, *i.e.*, oxidative stress, is closely related to neurodegenerative diseases.

ROS can also originate from microglia, which are likely to contribute to the degenerative course after activation. Microglia are the resident immune cells of the central nervous system (CNS), providing defense against external pathogens and pollutants and clearing of cellular debris. Activated microglia are present in diseased brains [8,9,10], indicating the contribution of inflammatory processes to neurodegeneration [11,12]. Chronic neuroinflammation, prolonged activation of microglia and astrocytes and persistent exposure to inflammatory cytokines are considered neurotoxic.

Excitotoxicity is the neuronal death caused by excessive or prolonged activation of excitatory amino acid receptors. The main participant in this process is glutamate, acting on ionotropic *N*-methyl-d-aspartate (NMDA), α-amino-3-hydroxy-5-methyl-4-isoxazole propionic acid (AMPA) and kainate receptors and on metabotropic glutamate receptors [3]. An excessive amount of glutamate in the synaptic cleft can result in the dysregulation of Ca^2+^ homeostasis, mitochondrial dysfunction and the generation of ROS and RNS. Under normal conditions, the presence and amount of glutamate is highly regulated, but in neurodegenerative diseases, this regulation is often disrupted, contributing to neuronal damage.

**Figure 1:**
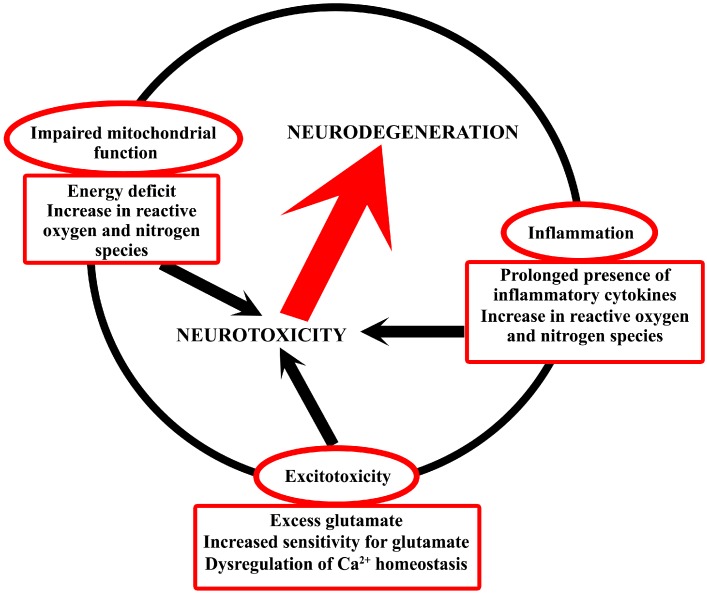
Schematic drawing of the common neurotoxic mechanisms in neurodegeneration. The figure attempts to interpret that the involvement and interrelationship of metabolic disturbances, neuroinflammation and excitotoxicity causes neurotoxicity that eventually results in neurodegeneration.

The mechanisms detailed above are in close relation in the development of neurodegenerative diseases [13,14,15] (Figure 1); however, their level of contribution to the pathological phenomena varies among the different disorders.

## 3. The Kynurenine Pathway

**Figure 2:**
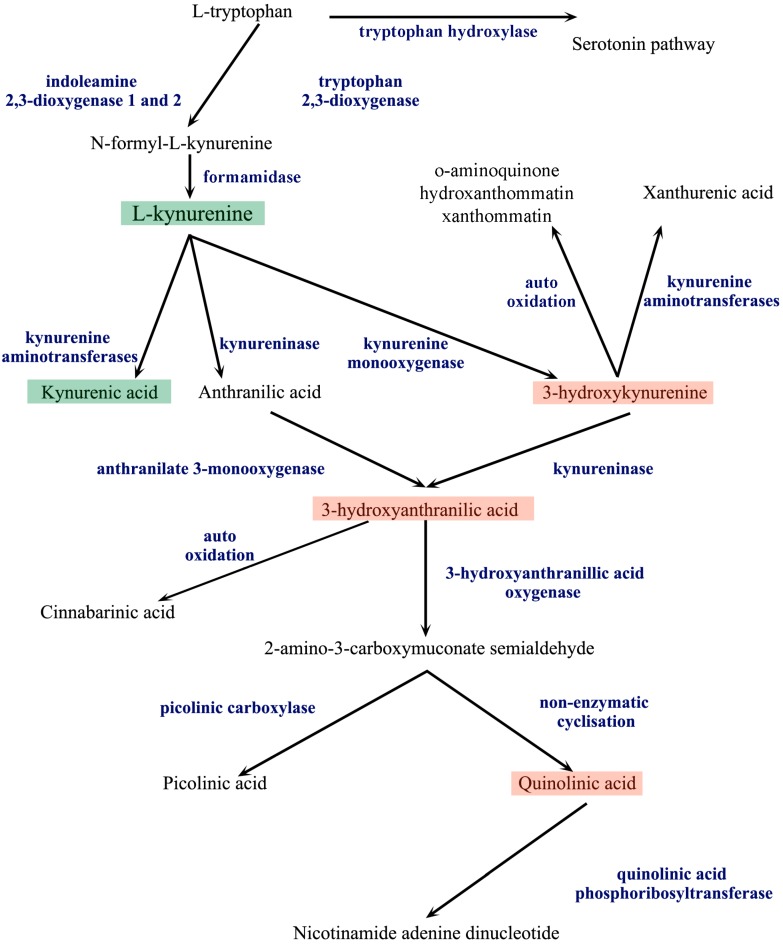
The kynurenine pathway. The metabolism of l-tryptophan is divided into two distinct pathways, the serotonin and the kynurenine pathway (KP). Indoleamine 2,3-dioxygenase 1 and 2 and tryptophan 2,3-dioxygenase convert l-tryptophan to *N*-formyl-l-kynurenine in the first step of the KP. *N*-formyl-l-kynurenine is further processed by formamidase to l-kynurenine (l-KYN), the central metabolite of the KP. From l-KYN, three different enzymes produce the next metabolites, forming three branches of the metabolism. The first branch is the kynurenic acid branch, where kynurenine aminotransferases (KATs) produce kynurenic acid from l-KYN. On the second branch, kynureninase converts l-KYN to anthranilic acid, which is further metabolized by anthranilate 3-monoxygenase to 3-hydroxyanthranilic acid (3-HA). On the third branch, kynurenine monooxygenase produces 3-hydroxykynurenine (3-HK), which is further metabolized by kynureninase to 3-HA. 3-HK can be also metabolized by KATs to form xanthurenic acid or be auto-oxidized. 3-HA is converted by 3-hydroxyanthranilic acid oxygenase to 2-amino-3-carboxymuconate semialdehyde or suffers auto-oxidation to form cinnabarinic acid. 2-amino-3-carboxymuconate semialdehyde can be converted by picolinic carboxylase to picolinic acid or can be converted by non-enzymatic cyclisation to quinolinic acid, which, through conversion by quinolinic acid phosphoribosyltransferase, results in the formation of nicotinamide adenine dinucleotide.

The main route of TRP metabolism is the kynurenine pathway (KP) (Figure 2), yielding neuroactive metabolites and nicotinamide adenine dinucleotide (NAD^+^). More than 95% of TRP is metabolized through the KP [16], while the remaining TRP is metabolized by the serotonin pathway.

In the first step of the KP, TRP is converted to *N*-formyl-l-kynurenine, an instable compound, by the enzymes tryptophan 2,3-dioxygenase (TDO) and indoleamine 2,3-dioxygenase 1 and 2 (IDO1, IDO2). These enzymes are the main rate limiting enzymes of the KP. Interferon-α, interferon-β (IFN-β) and interferon-γ (IFN-γ) are all able to induce IDO [17,18], but IFN-γ is considered as the main activator of IDO [19]. Furthermore, tumor necrosis factor-α and interleukin-6 are also able to activate IDO in an IFN-γ-independent way [20]. Previous investigations also showed that IDO has an important role in maternal-fetal tolerance, and both enzymes bear immunosuppressive properties [21,22].

*N*-formyl-l-kynurenine is further degraded by formamidase to l-kynurenine (l-KYN). l-KYN was long thought not to have neuroactive properties. l-KYN participation in oxidative processes has been proposed; pro-and also anti-oxidative properties have been documented [23].

Recently, evidence emerged that l-KYN is an endogenous ligand for the human aryl-hydrocarbon receptor (AHR) (Table 1) [24]. This receptor has an important role in cellular responses evoked by environmental toxins, such as 2,3,7,8-tetrachlorodibenzo-*p*-dioxin [25] and polycyclic aromatic hydrocarbons [26,27,28]. The AHR is a ligand-activated transcription factor, a member of the family of basic helix-loop-helix transcription factors [29]. It is a cytosolic protein that is normally inactive, but after ligand binding, it translocates to the nucleus, where it binds to the regulatory regions of xenobiotic-responsive elements. DNA binding indicates diverse transcriptional responses, such as the activation of enzymes participating in xenobiotic metabolism. AHR also participates in immune response [30,31] and in tumor genesis [32]. Besides environmental pollutants, numerous endogenous AHR ligands have been identified, including kynurenines l-KYN and kynurenic acid (KYNA) [33], suggesting the involvement of the KP in the diverse transcriptional pathways, regulated by AHR.

In the CNS, 40% of l-KYN is generated locally, and 60% is taken up from the blood [34]. The KP continues in different branches deriving from l-KYN, the first one leading to the synthesis of KYNA by kynurenine aminotransferases (KATs). KYNA is present in the human and rat brain in nanomolar concentrations [35], and it is an endogenous broad-spectrum antagonist of NMDA receptors acting at low concentrations on the strychnine-insensitive glycine site [36], while at higher concentrations on the glutamate recognition site (Table 1) [37]. Furthermore, it is a weak antagonist of kainate and AMPA ionotropic glutamate receptors [38]. At low concentrations (nM–μM), KYNA has a facilitatory effect on AMPA receptors, while at higher concentrations, it acts as a competitive antagonist [39,40]. The broad spectrum of KYNA’s receptorial action is in favor of its important role in regulation of glutamatergic neurotransmission.

On α7-nicotinic acetylcholine receptors (α7nAch), KYNA exerts a non-competitive antagonistic effect [41], thus participating in both glutamatergic and nicotinergic neurotransmission. Modulation of α7nAch, thus presynaptic glutamate release by KYNA, seems to be an important site of action in protection against glutamate induced excitotoxicity. KYNA has been proposed as an endogenous agonist of the G-protein coupled receptor 35 (GPR35) [42], the function of which is yet poorly elucidated. GPR35 is expressed in the gastro-intestinal system and in immune cells [42], but it is functional in dorsal root ganglia and also in hippocampal neurons [43,44]. It also has been demonstrated, that KYNA, similarly to l-KYN, is a ligand to AHR [33]. The effects of KYNA on GPR35 and AHR suggest an important immunomodulatory role for this compound. Recently, it was shown that KYNA is able to scavenge ROS in an NMDA- and nicotinic receptor-independent way; thus, it can be a potential endogenous antioxidant [45]. Taken together, KYNA is involved in possible neurotoxic processes as a protective agent, underlining its importance in neurodegenerative mechanisms.

The second branch of the KP deriving from l-KYN proceeds to anthranilic acid (AA) with the aid of kynureninase. AA has been shown to inhibit citric acid cycle and the respiratory chain complexes I–III [46], interfering with mitochondrial function. It may have an anti-inflammatory effect by forming a complex with copper and acting as an ^•^OH inactivating ligand [47,48]. Anthranilate 3-monooxygenase converts AA to 3-hydroxyanthranilic acid (3-HA), thus rallying to the third branch of l-KYN metabolism.

The first step of the third branch is the conversion of l-KYN by kynurenine monooxygenase (KMO) to 3-hydroxykynurenine (3-HK). KMO has the highest affinity for l-KYN among the enzymes of the three branches, suggesting that under physiological conditions, l-KYN is metabolized by this third branch [49]. 3-HK undergoes auto-oxidation, forming highly reactive o-aminoquinone and ROS—O_2_^•−^ and H_2_O_2_ [50,51,52,53]—and dimerizes to hydroxanthommatin and xanthommatin under physiological conditions [51,53]. 3-HK is generally considered as a neurotoxic agent *in vivo*, causing convulsive attacks when administered intraventricularly [54] or leading to tissue damage when administered intrastriatally [55]. 3-HK is also present in eye lens, and it has been connected with cataract formation [56].

The toxicity of 3-HK can be attributable to its capability to produce free radicals during its auto-oxidation. However, the free radical scavenging effects of this compound have also been observed *in vitro* in rat cortex and in C6 glioma cells [57]. Pro- or anti-oxidative properties of 3-HK under different circumstances were thoroughly reviewed by Colín-Gonzalez and colleagues [58].

The next step in the metabolism of l-KYN is the generation of 3-HA from either 3-HK by kynureninase or from AA by anthranilate 3-monooxygenase. 3-HA is also prone to auto-oxidation, generating superoxide radicals, H_2_O_2_, and cinnabarinic acid [59]. Cinnabarinic acid is a ligand for the type 4 metabotropic glutamate receptor and also for AHR [60,61]. 3-HA can induce apoptosis in monocytes/macrophages [62], and it can inhibit the mitochondrial respiratory chain [46,63]. Furthermore, it has important immunoregulatory functions by interfering with T-cell survival [64].

3-HK also can be transaminated by KAT to xanthurenic acid, which, similarly to 3-HK and 3-HA, possesses both pro- and anti-oxidative properties [65,66,67].

3-HA is further processed by 3-hydroxyanthranillic acid oxygenase (3-HAO) to 2-amino-3-carboxymuconate semialdehyde. This intermediate can be metabolized by picolinic carboxylase to produce picolinic acid (PIC) or can be transformed by nonenzymatic cyclisation to quinolinic acid (QUIN).

PIC is a non-selective metal ion chelating agent [68], induces morphological changes in the rat hippocampus, substantia nigra and striatum when administered intraperitoneally [69] and has a macrophage induction activity [70]. PIC is able to prevent QUIN-induced neurotoxicity when injected into the nucleus basalis magnocellularis of the rat [71], and it is able to modulate kainate-induced glutamate release from the striatum [72].

QUIN under normal conditions is present in the brain in nanomolar concentrations and metabolized for the synthesis of NAD^+^. *In vitro*, QUIN is toxic for brain cells from above 150 nM [73]. QUIN is a weak endogenous agonist on NMDA receptors [74], the action of which is selective, involving the receptor subtypes containing the NR2A and NR2B subunits [75]. QUIN causes the greatest excitotoxic damage in brain areas rich in NMDA receptors containing NR2A and NR2B subunits, mainly in the striatum and in the hippocampus [76]. Furthermore, it can increase glutamate release by neurons and inhibit glutamate uptake by astrocytes, maintaining an elevated level to constantly stimulate NMDA receptors, resulting in excitotoxicity [77]. Lipid peroxidation also contributes to QUIN toxicity [78]; results suggest that QUIN forms a complex with iron, and this complex can contribute to the formation of ROS [79,80]. The toxicity of QUIN on brain cells is exerted mainly through NMDA-mediated excitotoxicity [73,81].

**Table 1:** Short summary of the direct receptorial effects of kynurenine pathway metabolites.

Kynurenine Pathway Metabolite	Receptorial Effect	References
l-Kynurenine	Aryl hydrocarbon receptor (AHR) agonist	[24]
Kynurenic acid	NMDA receptor antagonist	[36,37]
	Dual effect on AMPA receptors: partial agonist at low nanomolar concentrations; antagonist at high micromolar-millimolar concentrations	[39,40]
	Kainate receptor antagonist	[38]
	α7-nicotinic acetylcholine receptor antagonist	[41]
	G-protein coupled receptor 35 agonist	[42]
	AHR agonist	[33]
Cinnabarinic acid	Type 4 metabotropic glutamate receptor agonist	[60]
	AHR agonist	[61]
Quinolinic acid	NMDA agonist	[74]

NMDA: *N*-methyl-d-aspartate; AMPA: α-amino-3-hydroxy-5-methyl-4-isoxazole propionic acid.

Quinolinic acid phosphoribosyltransferase converts QUIN to NAD^+^, finishing the metabolic process. NAD^+^ is thereafter utilized by different intracellular processes, serving as an electron transfer molecule.

The enzymes of the KP are differently distributed among the various cell types in the CNS, providing an important way of controlling the synthesis of different metabolites. Macrophages and microglia express the entire enzymatic machinery of the KP [82,83,84], and neurons are able to synthesize KYNA [85] and PIC [86], while astrocytes lack the enzyme KMO; therefore, they are not able to synthesize 3-HK under physiological conditions [83].

## 4. Kynurenines in Neurodegenerative Diseases

### 4.1. Alzheimer’s Disease

Alzheimer’s disease is characterized by progressive cognitive decline and memory loss, mainly in the elderly population. Pathological protein aggregates in the form of amyloid β (Aβ)-formed plaques, and phosphorylated tau (p-tau)-constituted neurofibrillary tangles are the main hallmarks of the disorder. An increased l-KYN/TRP ratio in AD patients suggests an enhanced TRP metabolism and the increased activation of IDO, which could be connected to neuroinflammation being activated by IFN-γ [87]. 3-HK levels are increased in peripheral blood of AD patients compared to controls [88], further supporting a metabolic shift in diseased patients. Furthermore, results showed that QUIN accumulates in the brain of AD patients, and it is co-localized with p-tau and neurofibrillary tangles [89,90]. Levels of KYNA are also elevated in AD patients, mainly in the striatum and in the hippocampus [91]. These result suggest that the kynurenine pathway in general is upregulated in AD, but the origin of this phenomenon needs further clarification. Activated microglia may produce an increased amount of KP metabolites in response to Aβ and p-tau, and it is possible that the excess amount of the produced QUIN leads to the invigoration of tau phosphorylation and to the development of a vicious circle [90].

### 4.2. Parkinson’s Disease

Parkinson’s disease is the second most prevalent neurodegenerative disease, characterized by pathological presence of Lewy bodies and Lewy neurites, comprised mainly of α-synuclein, in the neuromelanin-containing dopaminergic cells. Selective death of dopaminergic neurons is most abundant in the substantia nigra pars compacta; however, expansive neurodegeneration is present in the CNS [92]. The selective degradation of the nigrostriatal pathway leads to the motor symptoms characteristic of PD. Oxidative stress, excitotoxicity and neuroinflammation can both be significant factors in the selective death of dopaminergic cells [93,94]. The involvement of the KP in PD has been investigated in several studies. Widner and colleagues found an increased l-KYN/TRP ratio in serum and cerebrospinal fluid (CSF) of PD patients [95], while Ogawa and co-workers demonstrated decreased l-KYN and KYNA and increased 3-HK levels in brain samples of PD patients [96]. These result suggest the upregulation of TRP metabolism in PD, further supported by the finding that 3-HK levels increased in CSF [97].

### 4.3. Huntington’s Disease

Huntington’s disease is an autosomal dominantly inherited disorder caused by the CAG expansion in the HD gene on chromosome 4. This gene encodes the protein huntingtin, the normal functions of which are still under intensive research. The disease presents with motor symptoms and progressive cognitive decline. Excitotoxicity is considered as one of the main factors in the disease.

Administration of QUIN can serve as an animal model of HD, because it causes lesions pathologically similar to that of human samples, as it spares the cholinergic and aspiny neurons of the striatum [98]. Based on these observations, the quinolinate hypothesis of HD emerged, suggesting that QUIN may have a causative role in this disease. Several studies measured the levels of the KP metabolites, especially QUIN, in brain and CSF in postmortem samples of HD patients. Brain tissue levels of QUIN were found to be reduced [99]; also, the levels of KYNA were found to be decreased both in CSF and brain in HD [100,101,102,103], while an increase in 3-HK levels was found [104]. Measuring the same compounds during the early stages of HD revealed that both 3-HK and QUIN are elevated, while at later stages, no change or a decrease can be observed [105]. These results suggest that 3-HK and QUIN may be participants in the degenerative processes early in the course of HD.

### 4.4. Amyotrophic Lateral Sclerosis

ALS is a progressive neurodegenerative disorder, affecting motor neurons at both spinal, brainstem and cortical levels. ALS is mainly sporadic, with a 5%–10% occurrence of familial cases, affecting the adult population. About 20% of the familial cases is attributable to the mutation of the superoxide dismutase 1 (SOD1) gene [106], coding an important free radical scavenging enzyme. The symptoms include muscle weakness, atrophy and paralysis, and in most cases, the involvement of breathing muscles results in death within 3–5 years from disease onset.

Perturbations of the kynurenines in ALS were studied by several groups, Chen and colleagues found increased l-KYN and QUIN levels in CSF and serum of ALS patients [107], while KYNA levels were elevated in CSF of bulbar onset patients, but decreased in the serum of patients with severe clinical status [108].

The exact pathomechanism of ALS is not understood, but the general view is that it is a multifactorial disease, involving glutamate excitotoxicity, mitochondrial dysfunction, oxidative stress and inflammation. The kynurenines are involved in all of the above-mentioned processes, thus providing a multitarget option for therapeutic intervention in ALS.

### 4.5. Multiple Sclerosis

Demyelinization and the forming of sclerotic plaques are the most well-known characteristic of MS. The loss of the myelin sheath and the inflammation at different sites of the CNS causes diverse symptoms and distinct disease courses in MS. MS can be categorized based on the disease course as clinically-isolated syndrome, relapsing-remitting MS, primary progressive MS, secondary progressive MS and progressive relapsing MS.

Alterations in the KP were noted in MS; levels of TRP were found to be reduced both in the CSF and plasma of MS patients compared with control subjects [109,110]. Furthermore, KYNA levels were shown to be significantly decreased in CSF of MS patients during remission, while a significant increase is present both in the plasma and CSF in the course of relapse [111,112,113]. These findings suggest that levels of KYNA may be directly involved in the alternation of relapsing-remitting phases of the disease.

IFN-β treatment, a first line therapy applied in MS, has been shown to significantly increase l-KYN levels and the l-KYN/TRP ration, which is indicative of IDO activity. These results suggest that IFN-β treatment leads to the induction of IDO [114,115]; thus, the important role of IDO and the KP in immune response and autoimmunity is further confirmed (thoroughly reviewed in [4,116]).

### 4.6. Acquired Immunodeficiency Syndrome Dementia Complex

Infection with human immunodeficiency virus type 1 (HIV-1) can lead to the development of acquired immunodeficiency syndrome dementia complex (ADC), also termed HIV-associated dementia. ADC is a subcortical type of dementia characterized by marked memory impairment and psychomotor slowing. It occurs in the severe forms of HIV-1 infection, affecting about 2% of the infected population receiving combined antiretroviral therapy, as demonstrated by the CHARTER study [117]. Heyes and colleagues proposed that QUIN could play a direct role in the development of ADC, as QUIN levels were elevated in HIV-infected patients and correlated with the severity of neurological deficits [118]. A more feasible hypothesis suggests that QUIN is elevated due to the inflammatory process generated by HIV-1, nevertheless emphasizing the importance of the KP in inflammatory processes [119].

## 5. Therapeutic Perspectives

Neurodegenerative diseases are a great socio-economic burden, and in most cases, the therapies available provide only symptomatic relief; they do not treat the cause of the disorders. Consequently, there is a constant need for new and effective therapeutic solutions. Manipulating the KP could result in beneficial effects, affecting each of the contributory mechanisms of neuroinflammation, oxidative stress and excitotoxicity.

One of the possible beneficiary therapeutic interventions is the shifting of KP metabolism towards the formation of protective agents, mainly KYNA (Table 2). This metabolic shift can be achieved by specific enzyme inhibitors of KMO, kynureninase and 3-HAO. Several compounds have been developed for the inhibition of these enzymes [120,121,122], but the inhibitors of KMO have been the most widely studied and may provide the most beneficial effects, because inhibition of KMO prevents the formation of the most neurotoxic kynurenines, 3-HK, 3-HAA and QUIN.

**Table 2:** Therapeutic options for modulating the kynurenine pathway in neurodegenerative diseases, with some of the candidates developed and tested so far.

Enzyme Inhibitors	Kynurenic Acid Prodrugs or Analogs
3,4-dimethoxy-*N*-[4-(3-nitrophenyl)thiazol-2-yl]benzenesulfonamide (Ro-61-8048)	l-Kynurenine
2-(3,4-dimethoxybenzenesulfonylamino)-4-(3-nitrophenyl)-5-(piperidin-1-yl)methylthiazole (JM6)	Combination of l-kynurenine and probenecid *N*-(2-*N*,*N*-dimethylaminoethyl)-4-oxo-1H-quinoline-2-carboxamide hydrochloride
nicotinylalanine	7-Chlorokynurenic acid
	4-Chlorokynurenine (AV-101)

Inhibition of KMO leads to a decrease in the levels of 3-HK and QUIN in rats [123]. Blockade with 3,4-dimethoxy-*N*-[4-(3-nitrophenyl)thiazol-2-yl]benzenesulfonamide (Ro-61-8048), a potent KMO inhibitor, results in increased KYNA content in parkinsonian monkeys, and when combined with levodopa, it reduces the severity of dyskinesias, both acutely and after prolonged administration [124,125]. A prodrug of Ro-61-8048, 2-(3,4-dimethoxybenzenesulfonylamino)-4-(3-nitrophenyl)-5-(piperidin-1-yl)methylthiazole (JM6), was able to ameliorate anxiety-related behavior, spatial memory deficits and synaptic loss in a transgenic mouse model of AD and was able to decrease microglial activation and extend life span of R6/2 mice (a genetic mouse model of HD) [126]. However, the true prodrug properties of JM6 were questioned, and there is no doubt that Ro-61-8048 is an effective KMO inhibitor [127].

Another possibility for therapeutic intervention is the pharmacological increase of KYNA’s effect, either by administration of its prodrug or by synthetic analogs.

Combined administration of l-KYN and probenecid (PROB), an inhibitor of organic anion transport, causes elevation in the cortical levels of KYNA [128]. The same combination was shown to reduce the toxic effects of 6-hydroxydopamine, a widely-used model of PD in rats; joint administration was able to mitigate rotation behavior and neurodegeneration [129]. l-KYN administered together with PROB was able to attenuate histopathological changes and improve spatial memory in the Aβ model of AD [130]. Furthermore, l-KYN alone mitigated the neuronal cell loss and damage after ischemic insult in rats [131,132].

A combination of enzyme inhibition and pharmacological supplementation of KYNA also proved to be protective in a model of PD. l-KYN and PROB were combined with nicotinylalanine, an inhibitor of kynureninase and KMO, and were able to modulate QUIN-induced turning behavior [133].

**Scheme 1:**
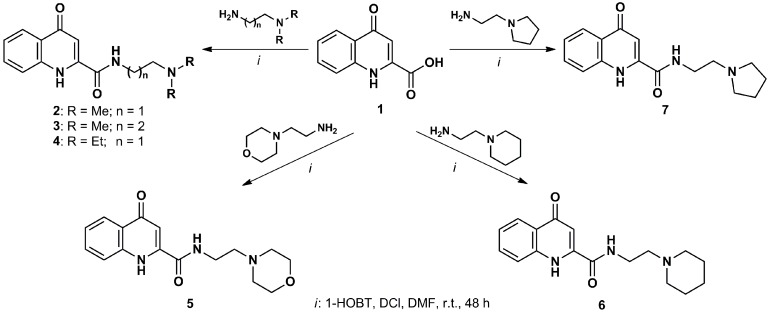
Transformational possibilities to develop kynurenic acid analogues. The transformations of kynurenic acid (KYNA) derivatives can be achieved through modification of the aromatic ring, the synthetically active 4-OH group, conversion of the 2-carboxylic function to pharmacologically interesting ester or amide derivatives of KYNA [139]. The amides of KYNA are pharmacologically and synthetically highly promising synthons in the patent literature. Coupling between KYNA and 2-dimethylaminoethylamine was achieved by using *N*,*N'*-diisopropylcarbodiimide (DCI) in the presence of 1-hydroxybenzotriazole hydrate (1-HOBT), yielding **2**. Further transformations are also shown in Scheme 1 [140].

During the past few years, several types of KYNA analogs have been synthesized (Scheme 1). A novel KYNA amide, *N*-(2-*N*,*N*-dimethylaminoethyl)-4-oxo-1H-quinoline-2-carboxamide hydrochloride, exerted beneficial effects in numerous paradigms. In micromolar concentrations, it reduced the amplitude of field excitatory postsynaptic potentials in the CA1 region of the hippocampus, while in nanomolar concentrations, it exerted a facilitatory effect, similar to KYNA [134]. In a transgenic mouse model of HD, the same analog increased survival time and prevented weight loss and striatal neuron loss of the animals [135]. The tests of the anti-inflammatory properties of this compound showed that it has a higher potency to inhibit tumor necrosis factor-α production than KYNA, suggesting a more potent immunoregulatory effect [136]. Similarly to KYNA, it also possesses protective characteristics against ischemia-induced neuronal loss [137]. The exact mechanism of action of this new compound is not known, but the lack of cognitive side effects makes it a promising candidate for further investigations [138].

Halogenated KYNA analogues have also been tested; 7-chlorokynurenic acid is a selective antagonist of the glycine site of NMDA receptors [141], and it was able to modulate kainate-induced neurodegenerative changes [142,143]. However, the blood-brain barrier permeability of this drug is not optimal; therefore, its prodrug, 4-chlorokynurenine (also known as AV-101), was examined and has been shown to be protective against QUIN neurotoxicity [143]. AV-101 has successfully completed a phase I clinical trial for the evaluation of safety, tolerability and pharmacokinetic profile (ClinicalTrials.gov Identifier: NCT01483846) and hopefully will be further processed.

In summary, the interventions affecting the KP are promising targets in the development of neuroprotective strategies.

## 6. Conclusions

The KP is an important target for the development of new therapies against neurodegenerative disorders, as it is comprised of compounds influencing processes related to excitotoxicity, oxidative damage and inflammation. Compiling evidence suggests that the interplay between the immune and nervous system in neurodegeneration could be the main target of both diagnostic and therapeutic developments. Therefore, further characterization of the role of KP metabolites in the immune and nervous system could be the key to new pharmaceutical therapies. However, owing to the broad effects of certain KP metabolites, a more complex approach would be necessary to develop drugs with the fewest side effects.

## Abbreviations

α7nAch: α7-nicotinic acetylcholine receptor
AA: anthranilic acid
Aβ: amyloid β
ADC: acquired immunodeficiency syndrome dementia complex
AD: Alzheimer’s disease
AHR: aryl-hydrocarbon receptor
AMPA: α-amino-3-hydroxy-5-methyl-4-isoxazole propionic acid
ALS: amyotrophic lateral sclerosis
CNS: central nervous system
CSF: cerebrospinal fluid
GPR35: G-protein coupled receptor 35
HIV-1: human immunodeficiency virus type 1
H_2_O_2_: hydrogen peroxide
HD: Huntington’s disease
IDO1: indoleamine 2,3-dioxygenase 1
IDO2: indoleamine 2,3-dioxygenase 2
IFN-β: interferon-β
IFN-γ: interferon-γ
KAT: kynurenine aminotransferase
KMO: kynurenine monooxygenase
KP: kynurenine pathway
KYNA: kynurenic acid
l-KYN: l-kynurenine
MS: multiple sclerosis
NAD^+^: nicotinamide adenine dinucleotide
NMDA: *N*-methyl-d-aspartate
^•^NO: nitric oxide
^•^NO_2_: nitrogen dioxide
O_2_^•−^: superoxide anion
^•^OH: hydroxyl radical
ONOO^−^: peroxynitrite anion
PD: Parkinson’s disease
PIC: picolinic acid
p-tau: phosphorylated tau
QUIN: quinolinic acid
ROS: reactive oxygen species
RNS: reactive nitrogen species
SOD1: superoxide dismutase 1
TDO: tryptophan 2,3-dioxygenase
TRP: tryptophan
3-HA: 3-hydroxyanthranilic acid
3-HK: 3-hydroxykynurenine
3-HAO: 3-hydroxyanthranillic acid oxygenase
